# The Effect of Sandblasting and Coating of Zirconia by Nano Composites on Bond Strength of Zirconia to Resin Cements

**DOI:** 10.30476/DENTJODS.2019.77789.0

**Published:** 2020-03

**Authors:** Parisa Karami Zarandi, Azamsadat Madani, Hosein Bagheri, Maryam Moslemion

**Affiliations:** 1 Dept. of Prosthodontics, Faculty of Dentistry, Zanjan University of Medical Science, Zanjan, Iran; 2 Dental Material Research Center, Mashhad University of Medical Science, Mashhad, Iran

**Keywords:** Sol-gel, Dip coating, µSBS, Resin cement, Zirconia

## Abstract

**Statement of the Problem:**

Despite yttria-stabilized tetragonal zirconia polycrystal (Y-TZP) high strength in dental restoration application, Zr- the framework has a low tendency to react chemically with cement which is the main reason of these restoration failures.

**Purpose:**

The aim of this in vitro study was to evaluate the effect of Y-TZP coating by nanocomposite of silica and aluminosilicate according to the sol-gel dip-coating technique on the bond strength of resin cement to Y-TZP.

**Materials and Method:**

In this experimental study, Y-TZP blocks (10×10×3mm^3^) were prepared and sintered and assigned into 4 groups (n=10) for coating including control group without any further surface treatment, sandblasted using 110μm alumina particles under 2.5 bar and tip distance of 10 mm, silica sol dip coating+calcination, aluminosilicate sol dip coating+ calcinations. To confirm chemical bonds of sol-gel covers, Fourier transforms infrared spectroscopy (FT-IR) technique was used. The surface of the sample was investigated by scanning electron microscopy (SEM), energy-dispersive spectroscopy detector (EDS) and x-ray diffraction (XRD) methods. Micro-shear bond strengths (µSBS) of zirconia-cement specimens were evaluated. Data were analyzed with a one-way ANOVA test in SPSS version 11.5 software with a confidence interval of 95%.

**Results:**

µSBS of sandblasting, nano-silica, and nano-aluminosilicate specimens were significantly higher than control. µSBS of nano-silica was higher than other groups but no significant difference was observed in µSBS of sandblasting nano-silica, and nano-aluminosilicate groups (*p*> 0.05).

**Conclusion:**

Covering the zirconia surface with non-invasive nano-silica and nano-aluminosilicate using the sol-gel technique leads to improved cement bond strength.

## Introduction

Surface preparation of zirconia-based dental restoration for resin bonding is still being considered as a major challenge. Unlike feldspathic porcelain and glass-ceramics, zirconia cannot be etched by hydrofluoric acid because its structure is completely made of crystalline phase and no glassy matrix exists. Therefore, a method other than acid etching must be used to provide microstructures for creating micromechanical adhesion or chemical bonding to improve the zirconia-resin bonding. 

So far, many researchers have examined different methods for creating zirconia surface roughness and improving zirconia-resin bonding [ 1 - 5 ]. Various methods were introduced including; application of phosphate monomers (MDP), special primers, formulation of new silane, applying low fusing porcelain, slurry zirconia and silica coating [ 6 - 12 ].

Different methods have been developed for silica coating on the zirconia surface, including silica pyrolytic coating, tribochemical coating, and plasma spraying techniques, Silano-Pen treatment, vapor-phase deposition, and coating using sol-gel methods [ 12 - 20 ].

According to reports on silica coating techniques, tribochemical is preferred due to improved bonding and ease of application. In the tribochemical coating technique, the bonding surface is air-abraded with silica-coated alumina particles. Aluminum oxide particles sprinkled with silica are buried on the ceramic surface by air pressure leading to a more chemically reactive surface to the resin. This method not only provides the silica surface for silanization but also causes micromechanical retention. However, literature shows‏ that sandblasting with silica-coated alumina particles causes stress on yttria-stabilized tetragonal zirconia polycrystal (Y-TZP) due to a decrease in degradation temperature of zirconia while the tetragonal phase transforms to monoclinic form [ 21 - 26 ].

In our previous study, we have reported the sol-gel dip coating method as a gentle, while the effective procedure for the formation of silicate and aluminosilicate thin films directly from the solution on the dental zirconia substrate to improve the bond strength of zirconia to the porcelain. To evaluate the coating characterization, Fourier transforms infrared spectroscopy (FT-IR), x-ray diffraction (XRD), scanning electron microscopy (SEM), and energy-dispersive spectroscopy detector (EDS) tests were used. The analysis of these tests confirmed the creation of a smooth and uniform layer of nanocomposite on the zirconia surface that improved the mechanical feature of the zirconia–porcelain bond after porcelain firing [ 27 ].

In this study, silica and aluminosilicate have been used through the dip coating sol-gel method for zirconia coating to improve the bond strength of zirconia to resin cement. The null hypothesis tested was that the coating of Y-TZP surface functionalization by different coatings has no effect on the resin-zirconia bond strength.

## Materials and Method

### Specimen preparation

Forty Y-TZP blocks (10×10×3mm3) were prepared (DD Bio ZW iso Zirconoxid; Dental Direkt GmbH) and sintered as instructed by the manufacturer. The sintered blocks were assigned into 4 groups (n=10) for subsequent coating including the control group without any further surface treatment, sandblasted using 110μm alumina particles under 2.5 bar and tip distance of 10mm, silica sol dip coating+calcination and aluminosilicate sol dip coating+calcinations. The compositions and manufacturers of materials used in this study are listed in Table 1.

**Table1:** Materials composition

Material	Specification of material	Manufacture
DD Bio ZW iso zirconoxid	Yttria-stabilized tetragonal zirconia polycrystal (ZrO_2_+HfO_2_+Y_2_0_3_>99%)	Dental Direkt GmbH, Germany
Clearfil TM SA luting	Dual cure resin cement (MDP, silane, silica and initiators)	Kuraray co., Okayama, Japan
Clearfil SE bond primer	Methacryloxydecyl dihydrogen phosphate, ethanol	Kuraray co., Okayama, Japan
Clearfil porcelain bond activator	Hydrophobic aromatic dimethacrylate, 3Methacryloxypropyl trimethoxysilane	Kuraray co., Okayama, Japan
Tetraethoxysilane	(C_2_H_5_O) _₄_Si	Merck, Germany
Aluminum nitrate nonahydrate	Al(NO_3_)_3_ · 9H_2_O	Sigma-Aldrich, USA
Isopropanol	CH_3_CH(OH)CH_3_	Merck, Germany

To provide a clean surface before coating, the blocks were washed under running distilled water, placed in an acetone/ethanol ultrasonic bath (Elmasonic S 300 H) for 20 minutes, and then air-dried at 70°C in Chamber Furnace - ELF11/6 [ 27 ]. 

### Sol preparation

The silica sol was prepared as described previously [ 28 ]. Tetraethylorthosilicate (TEOS) was added to nitric acid (HNO3) (≥65%, Sigma-Aldrich Co.) and ethanol (EtOH) 99.8% (Merck & Co., Inc) solution drop by drop. After 10 minutes of deionized water (DDW) was added to the solution, the obtained sol was stirred vigorously for 1hour. The molar ratio of EtOH: HNO3: EtOH: H2O was 1:1.7:6:2. Nano alumina-silica sol by Cao method [ 29 ] was prepared. Briefly, 67 ml of TEOS was dissolved in 100ml isopropanol. This solution was added dropwise to water and nitric acid at room temperature. This sol was stirred for 1hour. 7.36g aluminum nitrate nonahydrate, which was dissolved in 50ml deionized water, was added to partially hydrolyzed silica sol. This sol was stirred for 12hours.

### Coating of Y-TZP blocks

The Y-TZP blocks were coated by the sol using a dip-coating device and calcinated at 400°C (5°C/min) for 2 hours. The upward and downward motion speed was 80 mm/s and the dipping time was 60 seconds [ 27 ]. SEM, EDS, XRD and FTIR tests were accomplished to survey the coating layer and the findings of these experiments were expressed in our previous study [ 27 ].

### Coating characterization 

FT-IR, XRD, SEM and EDS were used to characterize the coating and the nature of the bonding between the coating and zirconia. The methods of these tests which we used in this study described in our previous article completely [ 27 ].

### Micro-shear- bond strength testing

**Figure1:**
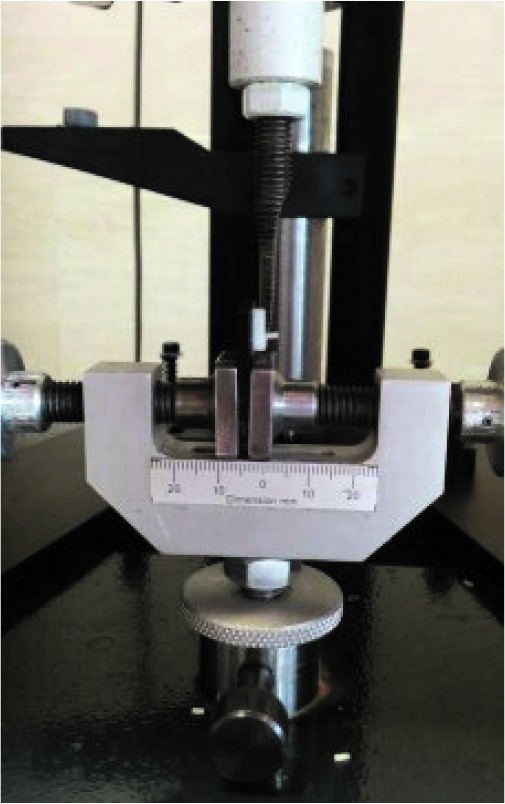
Universal testing machine instrument

To evaluate the efficacy of bonding between Y-TZP and resin cement µSBS testing was used. The coupling agent and primer (Clearfil, Kuraray) were thoroughly mixed according to the manufacturer instructions and applied on the Y-TZP block with a special brush. After placement, the whole surface was gently dried with oil- and water-free air spray. The resin cement pastes (Clearfil TM, SA, Kuraray, Japan) were mixed in a weight ratio of 1/1 and placed inside the Tygon tube with an internal diameter of 0.8mm and a height of 2mm. the Tygon tube was filled with cement and placed on the block, which was applied by coupling agent and primer. Then the excess cement was removed and the surface was exposed to LED curing light (Elipar II, 3M ESPE St. Paul, USA) in all directions by 1000 mW/cm^2^ intensity and 430-480 wavelength, for 40 seconds and stored in distilled water for 24 hours. The µSBS was tested with a universal testing machine instrument (STM20, SANTAM, Iran) with a crosshead speed of 1mm/ min (Figure 1).

The statistical results were analyzed using the one-way ANOVA test, which was followed by Tukey's post hoc pairwise comparison (α₌0.05) using SPSS V11.5 software.

## Results

The results of the analysis of the FT-IR, XRD, SEM, and EDS tests are explained in our previous study [ 27 ]. 

**Figure2:**
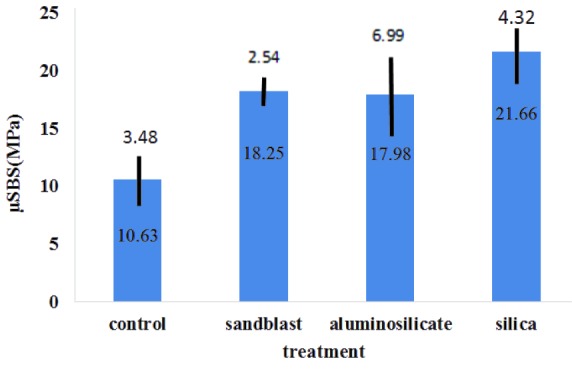
μSBS, micro shear bond strength)

The results of the µSBS test for each study group are shown in Figure 2. Statistical analysis revealed that there were significant differences between the study groups. The highest µSBS is observed for silica sol dip coated group (21.66±4.32 MPa) respectively and minimum bonding strength was observed in the control group (10.63±3.48 MPa). Tukey HSD post-hoc test showed a significant difference in the control group (10.63±3.48 MPa) and other groups. There was no significant difference between other groups (sandblasted: 18.25± 2.54 MPa, aluminosilicate: 17.98±6.99 MPa, silica: 21.66±32 MPa). The fracture mode in different groups was studied using a stereo microscope. The results showed that the fracture mode in all four groups was adhesive type.

## Discussion

The use of zirconia as a core in dental crowns improves fixed prosthesis due to high strength, tissue compatibility, and beauty.

However, one of the main disadvantages of the Zirconia framework is the low tendency to react chemically with cement and thereby its weak bond. Thus, various techniques and materials have been used for improving the bond between zirconia and resin cement [ 15 ]. The aim of the present study was to investigate the bond strength between zirconia and resin cement after the preparation of zirconia surface by silica or aluminosilicate sol-gel dip coating.

In this study, the results showed that the µSBS of the sandblasted samples and those coated with silica and aluminosilicate by sol-gel dip coating method was significantly higher than the control group. Therefore, the null hypothesis that there is no influence of Y-TZP surface treatment with sol-gel dip coating on the resin bond strength to Y-TZP is rejected. 

In the sol-gel dip coating method, the sol is first placed on the sample. Then, the solvent evaporates and nano-silica and nano-aluminosilicate gels are bonded to the zirconia surface through Van der Waals electrostatic forces. Finally, Si-O-Si bonds in silica and Al-O-Si bonds in aluminosilicate are reinforced by heat treatment and a solid layer binds to the surface. Then, hydroxyl groups (a chemical group with high polarity) in silica or aluminosilicate coated samples increase the surface polarity and reactivity of chemical bonds with other chemicals [ 30 ]. This reaction and formation of a new layer on the zirconia surface were confirmed by SEM, EDS, FTIR and XRD analysis in our previous study [ 27 ]. 

 The self-adhesive luting cement (Clearfil SA, Kurary,Japan) used in this study is an MDP-based silane containing resin cement. Previous studies showed that these characteristics have improved the bonding between resin cement and neutral surfaces like zirconia and alumina core surface or silica-based surface due to silanization [ 31 - 33 ]. Therefore, this cement was used for its high adhesive strength to Y-TZP ceramics. In this study, micro shear-bond strength of 21.66 MPa was reported for Clearfil SA after one-day storage in water, which were significantly higher than those obtained with the RelyX Unicem (3M ESPE;USA) and Variolink II (Ivoclar Vivadent, Liechtenstein) composite luting agents (19.9 and 15.2MPa) [ 14 - 15 ].

One of the common methods used to create micromechanical retention on the surface and increase bond strength is sandblasting with alumina powder. According to the results, sandblasting increases the bond strength of resin to zirconia. This increase can be due to micromechanical retention and the bond of hydroxyl groups of alumina and MDP monomer of resin cement [ 23 ]. Jevinkar P *et al*. [ 16 ] also used 110-µm alumina particle for sandblasting and found an increase in the cement bond strength to zirconia. However, sandblasting with alumina or silica-alumina (Cojet) might increase surface stress making crack formation and distribution during the time [ 23 - 25 ]. Some authors recommended sandblasting with particle size below 50µm, applying to sandblast at low pressure (1 to 2 bars) in order to minimize zirconia surface microcrack [ 26 ].

According to Chen C *et al*. [ 14 ], cement bond strength to zirconia in samples coated with nano-silica by sol-gel method was significantly higher than sandblasted samples. However, Lung C *et al*. [ 17 ] found that the bond strength of composite resin to zirconia prepared by sandblasting was higher than the samples, which were coated with silica by using sol-gel method. The differences observed, can be attributed to the method used by Lung C *et al*. [ 17 ] as they used sandblasting of silica-coated alumina particles.

Various studies show the reasonable performance of the sol-gel method in creating a layer of nano-silica. In our study, a silica or aluminosilicate network forms, through hydrolysis and polycondensation reaction. On the other hand, existing Si-OH and Al-OH have the potential to create a chemical bond to resin. As another advantage, in the sol-gel method, less working space and cheaper chemicals are used in comparison to expensive machines used for sandblasting. 

So far, the silica sol-gel coating technique been used to increase the bond strength to zirconia. This is the first study in which aluminosilicate particles are coated on the surface by a sol-gel dip coating method. According to literature, the zirconia surface has been mainly coated with nano-alumina. Külünk T *et al*. [ 18 ] found no increase in resin bond strength to zirconia coated with a layer of nano-alumina particles. The reactive magnetron sputtering method had been used in their study. On the other hand, Jevincar P *et al*. [ 16 ] and Zhang *et al*. [ 20 ] found an increase in resin bond strength after nano alumina coating by immersing the blocks in the AlN suspension. In this study, aluminosilicate coating improved the µSBS but this strength is less than sandblast and silica-coated groups, this is probably due to silica particles that fill the gaps between alumina particles and inhibit the resin tag formation, which is important in bond strength.

## Conclusion

According to the results of the present study, the use of nano-silica and aluminosilicate coatings on zirconia can increase the µSBS of resin cement to zirconia. Therefore, methods other than a mechanical procedure like thribochemical coating technique, which may have long destructive effects on the zirconia- resin cement bonding, could be used.
